# Cigarette Smoking and Effects on Hormone Function in Premenopausal Women

**DOI:** 10.1289/ehp.7899

**Published:** 2005-06-03

**Authors:** Gayle C. Windham, Patrick Mitchell, Meredith Anderson, Bill L. Lasley

**Affiliations:** 1 Division of Environmental and Occupational Disease Control, California Department of Health Services, Oakland, California, USA; 2 California Department of Health Services, Sacramento, California, USA; 3 Impact Assessment Inc., La Jolla, California, USA; 4 Institute of Toxicology and Environmental Health, School of Medicine, University of California, Davis, California, USA

**Keywords:** cigarette smoking, endocrine disruption, estrogen, follicle-stimulating hormone, hormones, menstrual dysfunction, progesterone, steroids, women’s health

## Abstract

Cigarette smoke contains compounds that are suspected to cause reproductive damage and possibly affect hormone activity; therefore, we examined hormone metabolite patterns in relation to validated smoking status. We previously conducted a prospective study of women of reproductive age (*n* = 403) recruited from a large health maintenance organization, who collected urine daily during an average of three to four menstrual cycles. Data on covariates and daily smoking habits were obtained from a baseline interview and daily diary, and smoking status was validated by cotinine assay. Urinary metabolite levels of estrogen and progesterone were measured daily throughout the cycles. For the present study, we measured urinary levels of the pituitary hormone follicle-stimulating hormone (FSH) in a subset of about 300 menstrual cycles, selected by smoking status, with the time of transition between two cycles being of primary interest. Compared with nonsmokers, moderate to heavy smokers (≥ 10 cigarettes/day) had baseline levels (e.g., early follicular phase) of both steroid metabolites that were 25–35% higher, and heavy smokers (≥ 20 cigarettes/day) had lower luteal-phase progesterone metabolite levels. The mean daily urinary FSH levels around the cycle transition were increased at least 30–35% with moderate smoking, even after adjustment. These patterns suggest that chemicals in tobacco smoke alter endocrine function, perhaps at the level of the ovary, which in turn effects release of the pituitary hormones. This endocrine disruption likely contributes to the reported associations of smoking with adverse reproductive outcomes, including menstrual dysfunction, infertility, and earlier menopause.

Cigarette smoke contains known reproductive toxicants, and smoking has been associated with adverse reproductive outcomes in women such as infertility, subfecundity, younger age at menopause, and menstrual disorders (Department of Health and Human Services 2001). Previously (Windham et al. 1999), we reported that smokers had different menstrual cycle characteristics compared with nonsmokers; heavy smoking was associated with shorter and more variable cycle lengths, with the shortening occurring primarily during the follicular phase. There was some suggestion of increased risk of short luteal phase (< 11 days) and anovulation as well, but the confidence intervals were quite wide. The mechanism of these and other reported effects is not known but may reflect alterations in hormone function by components of tobacco smoke, with smoking suggested as having antiestrogenic effects (Baron et al. 1990). However, studies examining estrogen and its metabolites by smoking status have found somewhat mixed results (Berta et al. 1992; Key et al. 1996; Longcope and Johnston 1988; Zumoff et al. 1990), perhaps partly due to limited sampling points.

Hormone function is difficult to study in non-clinic-based populations because of the cyclical nature of excretion and day-to-day variation in premenopausal women. Under the control of the complex hypothalamic–pituitary–ovarian (HPO) axis, the steroids estrogen and progesterone are released from and reflect ovarian activity and in turn modulate release of gonadotropins from the pituitary via a negative feedback loop. Rising levels of the pituitary hormone follicle-stimulating hormone (FSH) during the luteal to follicular phase transition between menstrual cycles are critical for follicle recruitment and development in the subsequent cycle (van Santbrink et al. 1995). Declining levels during the follicular phase are important for selection of a dominant follicle and its maturation, and FSH peaks again around ovulation. FSH level is considered a marker of ovarian reserve or fertility (Scott et al. 1989; Scott and Hofman 1995) and is elevated among women approaching menopause (Backer et al. 1999; Burger et al. 1995). As such, it may also be useful for identifying ovarian toxicants (Marcus et al. 1993). A few studies have suggested that smokers have higher levels of FSH than do non-smokers (Backer et al. 1999; Cooper et al. 1995; Cramer et al. 1994). These studies were based on a single serum sample, collected either very early in the cycle or with the cycle timing unknown, and tended to include older women, some of whom were perimenopausal or postmenopausal. Therefore, the purpose of the present study was to examine smoking in relation to the patterns of urinary hormone metabolites throughout the menstrual cycle in premenopausal women to determine whether smoking may exert some of its deleterious effects via an endocrine mechanism. In particular, we were interested in measuring whether estrogen excretion appeared reduced and identifying at what level such an effect may occur by examining the pituitary gonadotropin FSH. Ours is the first study to examine hormone dynamics, with daily metabolite levels of the steroids throughout the cycle and FSH during the luteal–follicular transition, in relation to smoking that was verified by bioassay.

## Materials and Methods

The data for this investigation are derived from the Women’s Reproductive Health Study, a prospective study of menstrual function and early pregnancy loss conducted among 403 premenopausal women, whose data collection and analytic methods have been described previously (Waller et al. 1998; Windham et al. 1999) and are summarized briefly below. The institutional review boards of both Kaiser Permanente Medical Group and the California Department of Health Services approved the protocol, and participants provided written informed consent.

Women 18–39 years of age enrolled in the Kaiser Permanente Medical Care Program in northern California during 1990–1991 were screened by a short telephone interview to determine eligibility (based on possibility of becoming pregnant) and willingness to collect and freeze first morning urine samples daily for up to 6 months (Waller et al. 1998). About half of those eligible agreed to participate, but some later dropped out (16%) or became ineligible (11%), leaving 403 women who completed urine collection. On average, women collected urine on 92% of appropriate study days during 5.6 menstrual cycles, but because urine collection was not timed to the cycle start dates, a mean of 3.6 complete cycles were collected per woman. Steroid metabolites were measured daily, and FSH was measured in a subset of 300 cycles after additional funding was obtained. Participants completed a detailed baseline telephone interview that asked about demographics, reproductive history, lifestyle factors, and various exposures, including past and current cigarette smoking. Women filled out daily diaries during urine collection, recording vaginal bleeding, intercourse, and contraception, as well as the number of cigarettes smoked each day.

### Smoking assessment.

The diary was used for determining amount smoked because the daily levels reported provided cycle-specific measures. The average number of cigarettes smoked per day for each cycle was calculated and then categorized; for the FSH subset, we primarily used none, low (1–9 cigarettes/day), and moderate smoking (≥ 10 cigarettes/day). In the full data set with larger numbers, we also delineated a heavy smoking category (≥ 20 cigarettes/day). To validate self-reported smoking, pooled urine samples of 5 days from two to three cycles of each woman were assayed for nicotine and its metabolite cotinine (Elkin et al. 1999; Windham et al. 1999). All of the nonsmokers had urinary cotinine levels < 25 ng/mL, a cut point well within the range used in other studies (Benowitz 1996). Two women who reported less than daily smoking, but with measured cotinine levels > 200 ng/mL, were excluded as potential misreporters, as was a nonsmoker who used nicotine gum.

### Hormone end points.

#### Steroids.

The primary estradiol metabolites estrone sulfate and estrone glucuronide [estrone conjugates (E1C)] and the progesterone metabolite pregnanediol-3-glucuronide (PdG) were measured daily by enzyme-linked immunoassays and then adjusted for creatinine concentration, as described previously (Munro et al. 1991; Waller et al. 1998). We determined ovulatory status for each cycle based on a relative rise in progesterone above baseline levels (Kassam et al. 1996; Waller et al. 1998). The day of ovulation (or luteal transition) was estimated using a previously validated algorithm based on the ratio of E1C to PdG during 5-day windows where E1C was declining and PdG was increasing (Baird et al. 1990; Waller et al. 1998; Windham et al. 2002). In a small proportion (5.6%), we reassigned the day of ovulation to better correspond to individual steroid hormone plots. The cycle was divided into the follicular phase (calculated from the first day of menses through the estimated day of ovulation) and the subsequent luteal phase (day after ovulation to day before next menses). Cycle and phase lengths were categorized as short and long based on the 5th and 95th percentiles of their distributions (Windham et al. 1999). For examining and graphing mean daily values of the steroids by smoking status, the cycles were centered at the estimated day of ovulation.

As noted above, urine was collected during many partial cycles, so we generally excluded these, as well as cycles where ≥ 30% of days of urine were missing midcycle (~ 700 total). We only examined steroid parameters in the remaining 1,560 cycles for which a day of ovulation was assigned, so we could identify the follicular and luteal phases. Some incomplete first cycles are included in the analyses of luteal-phase measures if there was at least 20 days of urine collection and urine collection started within 14 days of the reported last menstrual period (*n* = 112). Cycles with incomplete luteal phases (which would include successful pregnancies) were not included in analyses of luteal-phase parameters, but 89 met the inclusion criteria for follicular-phase parameters (including 39 pregnancies). The primary hormone parameters calculated (Windham et al. 2002) for 1,451 follicular phases and 1,459 luteal phases include the following:

- Baseline: the E1C baseline value calculated as the mean over the first 5 days of the cycle. To avoid including elevated “spillover” progesterone values from the previous luteal phase or rising levels of the next, the PdG baseline is the minimum 5-day average occurring before the luteal-phase 5-day maximum.
- Daily average: Mean E1C and PdG levels calculated over the follicular phase or the luteal phase.
- Area under the curve, or “total”: the sum of the daily E1C values during the follicular phase or the sum of daily PdG values during the luteal phase.
- Peak: A 3-day average around the maximum E1C or PdG value. For the estrogen metabolite, the maximum value was selected within a 6-day window around the day of ovulation to capture the periovulatory peak. For progesterone, the maximum value during the luteal phase was selected. If values were missing for any of the 3 days, the peak variable was not calculated (18–20% of cycles).

#### Follicle-stimulating hormone.

After procuring additional funding, cycles were selected 2 years later for FSH assay, based on smoking status, to reach a goal of 300 cycles in total. Initially, all cycles of smokers (defined as an average of ≥ 1 cigarette reported/day or cotinine > 25 ng/mL) were selected, and two contiguous cycles of nonsmokers (cotinine ≤ 0.5 ng/mL) were randomly selected, targeting cycles early in urine collection to correspond to the timing of a saliva sample. The FSH assay, based on heat dissociation of the FSH heterodimer and measurement of the FSH β-subunit (Qiu et al. 1998), was conducted blind to smoking status at the University of California, Davis, laboratory where it was developed. Previous studies from the laboratory have shown good correspondence between the circulating FSH heterodimer and the urinary β-subunit (Li et al. 2002). The increase in FSH during the transition between menstrual cycles (e.g., luteal phase of one cycle to follicular phase of the next) is the initiator of events leading to ovulation, so this was identified as the time period of primary interest. FSH was measured in daily samples from 7 days before the first bleed day of a cycle through 17 days afterward to catch the periovulatory rise. Samples were run in duplicate and the average value used, unless the duplicates varied by > 20%, in which case they were rerun. FSH values were also creatinine adjusted. For final analyses, we used only cycles that had adequate specimen remaining to measure FSH during the time period of interest. If there was inadequate sample for nonsmokers, other cycles were substituted, but these were not available for smokers, so the final sample with FSH measures included 112 menstrual cycles among 32 smokers (9 smokers did not have sufficient urine for FSH analyses) and 209 cycles among 93 non-smoking comparisons.

Because there had been few prior studies examining daily FSH levels, we calculated the daily mean and the slope values for seven different 4–8 day windows of time within the luteal–follicular transition, which were consistent with earlier work from the laboratory (De Souza et al. 1998; Qiu et al. 1997). For FSH analyses, the first bleed day of the cycle was considered day 1 (with no day zero), and we primarily report results for the following windows: days −7 to −1, −5 to 1, −3 to 1, and −3 to 3. For comparison with other studies based on serum FSH, we also examined a basal level as the mean of days 1 to 5. We also examined the maximum FSH value midcycle (periovulatory) and the cycle day on which it occurred (within days 6–17). An FSH parameter was considered missing for a cycle if 25–30% of values within the window were missing (e.g., > 1 day of 4-day windows or 2 days of 7-day windows). This eliminated about 10% of sampling periods.

### Statistical analysis.

Numerous covariates from the baseline interview were examined as potential confounders, including demographics, reproductive history, body mass index (BMI), and other lifestyle factors (caffeine and alcohol consumption, physical activity). In a previous report, we identified variables associated with the steroid parameters (Windham et al. 2002) and examined these as categorical variables (Table 1) in relation to FSH level and smoking status by analysis of variance. Because the primary analysis is at the cycle level and a woman’s cycles are not independent observations, we used mixed models that account for repeated measures for multivariate modeling (Laird and Ware 1982; Zeger and Liang 1986), effectively increasing the standard error of the estimates. Variables identified as potential confounders were included in regression models with each covariate removed one at a time to determine if the association between smoking and a selected FSH parameter (slope or mean of days −5 to 1) changed. If so, that variable was included in final multivariate models. Similar methods were used to build models for examining smoking in relation to steroid levels. In final regression models, we weighted the hormone parameters by the proportion of nonmissing values in the appropriate time frame during each cycle (Windham et al. 2002). Thus, if there were no missing values, the weight was 1, with missing values resulting in down-weighting; mean weights for each parameter ranged from 0.77 to 0.91.

## Results

Overall, participants were predominantly white (71%), well educated (40% had a college degree), and parous (88%), with a mean age (± SD) of 31 ± 4.2 years (Waller et al. 1998; Windham et al. 2002). On the baseline questionnaire, 9.2% of women reported being current regular smokers, and the daily diaries indicated that 10% smoked an average of ≥ 1 cigarettes/day, with 5% smoking less frequently. Compared with nonsmokers, smokers were significantly less educated, more likely to be of a race other than Asian or white, drank more alcoholic and caffeinated beverages, and were more likely to have had any pregnancy and to have had a pregnancy loss or therapeutic abortion.

The characteristics of the FSH subset are shown in Table 1; smokers did not vary much from those in the overall study except they were even less likely to have a college degree. Nonsmokers in the subset were less likely to be Hispanic (p = 0.04) and more likely to be older (p = 0.10) than were all nonsmokers. The distribution of cycle characteristics (short cycle, long follicular phase, etc.) was similar in the subset and the overall study, and the association of shorter cycle length with smoking was also observed [crude risk ratio = 2.1; 95% confidence interval (CI), 1.1–4.0].

### FSH findings.

Among nonsmokers, mean FSH levels (for days −5 to 1) tended to be higher in nonwhite, older women with greater caffeine consumption, and levels were lower among women with a history of pregnancy loss and with greater alcohol consumption (Table 1). Education, BMI, and other reproductive history variables were not strongly associated with FSH levels. All the mean FSH parameters during the luteal–follicular phase transition were significantly inversely associated with the length of the next cycle and follicular phase, either adjusting for smoking status or among nonsmokers only. The slope parameters that included 5–7 days of the previous cycle were also inversely associated with cycle length.

**Figure 1:**
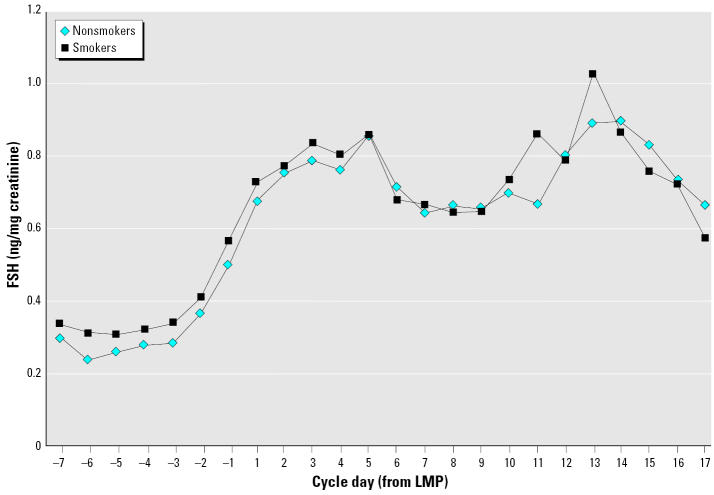
Mean daily levels of urinary FSH (ng/mg creatinine) by smoking status during the luteal–follicular phase transition. Day 1 is the first bleed day of a cycle, or the last menstrual period (LMP); negative days are in the previous cycle. Smokers exclude one woman with very high FSH values (see “FSH findings”); with her included, differences would be greater and extend farther into days 7–12. Data from Women’s Reproductive Health Study, California Department of Health Services.

As shown in Figure 1, the urinary FSH levels reflect the rise during the luteal–follicular transition, with the highest levels occurring early in the follicular phase at days 3–5 and then again around ovulation. Smokers tended to have higher daily FSH levels than non-smokers during the luteal–follicular phase transition period. Consistent with that, mean FSH parameters in moderate to heavy smokers (≥ 10 cigarettes/day) compared with non-smokers were statistically significantly increased for all seven of the original time windows (and including the early follicular-phase mean) examined. Light smokers had levels more similar to nonsmokers or very slightly lower. The slopes of FSH during the corresponding periods did not appear to vary consistently by smoking level. Smokers did tend to reach the midcycle peak FSH level > 1 day earlier than did nonsmokers, with an intermediate value among light smokers, but this was less strong after adjustment. One woman had very high FSH levels, which were confirmed on reassay; she was older, a moderate smoker, and a heavy caffeine consumer. She was excluded to determine the degree to which her values were influencing results. This reduced the mean differences between moderate smokers and nonsmokers by about half, so we present these to be conservative in Table 2 showing adjusted differences. Adjustment (for age, race, pregnancy history, BMI, and alcohol and caffeine consumption) changed the magnitude of differences in means only very slightly but increased the estimates for difference in slope parameters among moderate smokers. In general, when including the woman with the high FSH values, the mean FSH levels during the cycle transition were significantly elevated by 52–57% in moderate smokers, whereas excluding her, the means were elevated about 30–35% (Table 2). The effect of excluding her was not consistent for the FSH slope parameters.

Limiting the analyses to only average length cycles (25–35 days) slightly strengthened the elevations in FSH associated with moderate smoking. We examined the smoking level in the previous cycle as well, that is, the one that included the luteal phase of interest, because this would precede FSH measurements. A few cycles drop out because the smoking data are missing, but results were very similar, presumably because smoking habits did not vary greatly across cycles.

### Steroid findings.

**Figure 2:**
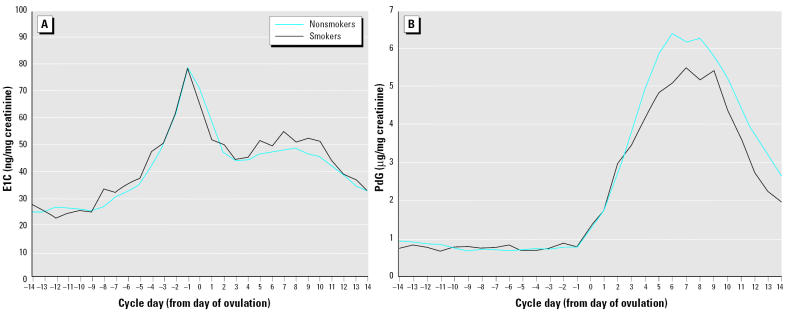
Mean daily levels of urinary E1C (ng/mg creatinine; A) and PdG (μg/mg creatinine; B), by smoking status, in one representative cycle per participant. Cycles are centered on the estimated day of ovulation (labeled day 0), so negative days are in the follicular phase and positive days are in the luteal phase. Data from Women’s Reproductive Health Study, California Department of Health Services.

Figure 2, of daily E1C and PdG mean levels, shows the characteristic cyclic patterns of estrogen and progesterone secretion. The steroid metabolites were examined in separate models that included age, race, education, pregnancy history, metabolic equivalence (MET) score (exercise levels), and caffeine consumption. The baseline levels of both steroid hormones were elevated among the heaviest smokers in multivariate models, but not statistically significantly: 22% for E1C and nearly 40% for PdG (Table 3). These elevations were significant at the moderate smoking cut point (≥ 10 cigarettes/day), where there is more power; the mean baseline E1C was elevated > 25% (β = 6.3; 95% CI, 0.40–12.3), and baseline PdG was increased about 35% (β = 0.20; 95% CI, 0.03–0.38), compared with nonsmokers. Progesterone metabolite levels during the luteal phase were somewhat lower among smokers in general (Figure 2B) and consistently about 25% lower among the heaviest smokers (Table 3).

## Discussion

The present analysis showed that moderate to heavy smokers had elevated baseline (e.g., early follicular phase) levels of the steroid metabolites and heavy smokers had somewhat dampened progesterone metabolite levels during the luteal phase. Further, we found that mean urinary FSH levels during the time of the luteal–follicular phase transition were higher among moderate to heavy smokers than among nonsmokers. Combined with our previous findings of shorter cycle and follicular-phase lengths among heavy smokers (Windham et al. 1999), an alteration in the endocrine pattern with smoking is indicated.

Because of the nature of its association with various hormonally related diseases, smoking has been considered potentially anti-estrogenic. However, only a few studies have provided metabolic evidence to support this, and these studies are hampered by having few biosampling points, a small number of subjects, or inclusion of postmenopausal women. MacMahon et al. (1982) reported reduced urinary excretion of estrone, estradiol, and estriol in the luteal phase among smokers, suggesting that this may be due to reduced estrogen production. Michnovicz et al. (1986) found that smoking induced the 2-hydroxylation of estrone to relatively inactive metabolites and decreased excretion of estriol. However, several studies have not reported differences in serum estradiol concentrations with smoking in premenopausal women (Berta et al. 1992; Key et al. 1996; Longcope and Johnston 1988; Zumoff et al. 1990).

Some of the disease patterns observed with smoking may actually reflect increases in androgens or progesterone. A few studies have reported that smoking increases adrenal activity, with elevations in adrenal androgens seen mostly among postmenopausal smokers (Baron et al. 1995; Friedman et al. 1987; Key et al. 1991; Khaw et al. 1988). Zumoff et al. (1990) measured serum levels at multiple points during the cycle and reported elevated serum progesterone levels during the early follicular phase among smokers, when most progesterone is of adrenocortical origin. This is consistent with our finding of elevated baseline progesterone levels among heavier smokers. However, those authors did not report differences in progesterone levels during the luteal phase. Estrogen was increased in the follicular phase among smokers in that study, which we tended to observe as well. Similar to our finding, Berta et al. (1992) found that regular moderate smokers (≥ 10 cigarettes/day for at least 5 years) had lower plasma progesterone levels on a single sample day during the midluteal phase. With an increased baseline PdG reflecting more progesterone of adrenal origin in smokers, the decreased luteal-phase PdG levels we observed may indicate even lower corpus luteum contribution of progesterone to total excretion. Some in vitro studies (Bodis et al. 1992; Gocze et al. 1999; Miceli et al. 2005) have found inhibition of progesterone production by granulosa cells or luteal cells that were treated with cigarette smoke extract or the alkaloids found in smoke (e.g., nicotine, cotinine, anabasine).

The serum FSH level during the first 3–4 days of the cycle is useful clinically to assess fertility and predict success of in vitro fertilization, as well as to identify the perimenopausal transition (Burger et al. 1995; Mausher et al. 1988; Scott et al. 1989). The few other studies that examined FSH in relation to smoking were based on single serum samples and included women at older ages when FSH may be increasing perimenopausally. Two studies that measured FSH at the beginning of the cycle found higher levels associated with smoking (Cooper et al. 1995; Cramer et al. 1994), as did a study in which the timing of the serum draw was not known (Backer et al. 1999). These studies support our findings of elevated FSH levels with smoking, but our results expand upon them by examining the dynamics, showing that the elevation in FSH levels among smokers is observable at the end of the prior luteal phase. Furthermore, we observed this effect among reproductive-age women, before onset of the perimenopausal transition.

There are some limitations of the present study that should be considered. Women who comply with the labor-intensive urine collection protocol may not be entirely generalizable, and the eligibility criteria would tend to exclude women with chronic menstrual cycle disturbances. We measured estrone metabolites, which may vary by woman in how well they reflect serum estrogen levels. Furthermore, we cannot establish whether secretion or metabolism is affected by smoking. Our power was somewhat limited for examining FSH levels, because of limited funding and inadequate remaining urine sample for some participants. Thus, for example, we could not examine heavier smoking levels in relation to FSH. We did not examine the effects of passive smoking in this study. The FSH subset should exclude most women exposed to environmental tobacco smoke (ETS) from nonsmokers based on the cotinine level criteria we used (< 0.5 ng/mL), but they would be included in steroid hormone analyses. If ETS causes effects in the same direction as active smoking, but presumably to a lesser extent, this would tend to dilute effects we observed because of ETS exposure being included in the comparison group. Therefore, our results may underestimate the magnitude of the true association with steroid levels.

In conclusion, the present data are consistent with some previously published reports but extend them and present for the first time the effect of smoking on steroid and gonadotropin patterns across cycles. This approach permits the evaluation of the integrity of the HPO axis during the entire period of follicular recruitment and maturation rather than just analyzing hormone patterns during individual menstrual cycles. Because progesterone modulates FSH in the endocrine feedback loop, the lower progesterone metabolite levels in smokers during the luteal phase are consistent with decreased entrainment of FSH during the luteal–follicular phase transition, leading to the elevations we observed. The shortening of the follicular phase may be a direct consequence of the increased FSH, consistent with other findings (Cramer et al. 1994; De Souza et al. 1998). The increase in FSH may accelerate the recruitment and development of follicles, moving ovulation earlier. Short follicular phase has been associated with decreased fecundity or in vitro fertilization rates in several studies (Check et al. 1992; Fukuda et al. 2001; Kolstad et al. 1999; Liss et al. 2002). Shorter follicular phase may result in inadequate follicle development, followed by inadequate corpus luteum function. Because progesterone controls endometrial response, it is critical for early pregnancy maintenance; luteal-phase deficiency or decreased progesterone has been implicated as a cause of infertility and fetal loss (Pittaway et al. 1983; Tulppala et al. 1991; Wuttke et al. 2001). This pattern of higher FSH levels and shorter cycles in smokers is also consistent with the observation that smokers tend to experience earlier menopause (Cooper et al. 1999; Midgette and Baron 1990). Thus, the decreased progesterone and perturbation of FSH suggest both a target and one mechanism by which cigarette smoke may alter ovarian function and reduce female fertility. Because cigarette smoke contains thousands of chemicals, this pathway may serve as a model for some endocrine effects of other environmental exposures.

## Figures and Tables

**Table 1:** Participant characteristics and mean FSH level in FSH subset, by smoking status.

	Nonsmokers		Smokers	
Variable	No. of women (%)	FSH (mean ± SD)^a^	No. of women (%)	FSH (mean ± SD)^a^
Race*				
White	72 (77.4)	0.36 ± 0.17	24 (75.0)	0.50 ± 0.48
Asian	15 (16.1)	0.51 ± 0.35	2 (6.3)	0.90 ± 0.50
Other	6 (6.5)	0.48 ± 0.20	6 (18.8)	0.32 ± 0.14
Age (years)				
< 30	26 (27.9)	0.33 ± 0.22	12 (37.5)	0.36 ± 0.14
30–34	34 (36.6)	0.42 ± 0.20	12 (37.5)	0.40 ± 0.21
≥ 35	33 (35.5)	0.42 ± 0.22	8 (25.0)	0.81 ± 0.74
Pregnancy history				
0 pregnancies	7 (7.5)	0.49 ± 0.38	1 (3.1)	0.46 ± 0.21
≥ 1 pregnancy, 0 losses	59 (63.4)	0.41 ± 0.22	20 (62.5)	0.52 ± 0.55
≥ 1 pregnancy, ≥ 1 loss	27 (29.0)	0.34 ± 0.15	11 (34.4)	0.45 ± 0.22
Education*				
No college	21 (22.6)	0.44 ± 0.23	16 (50.0)	0.55 ± 0.62
Some college	29 (31.2)	0.38 ± 0.16	13 (40.6)	0.43 ± 0.22
College graduate	43 (46.2)	0.38 ± 0.25	3 (9.4)	0.53 ± 0.14
BMI (kg/m2)				
< 19.1	6 (6.5)	0.47 ± 0.44	2 (6.3)	0.29 ± 0.08
19.1–27.3	66 (71.0)	0.39 ± 0.21	19 (59.4)	0.58 ± 0.54
> 27.3	21 (22.6)	0.38 ± 0.18	11 (34.4)	0.41 ± 0.31
MET score				
0	42 (45.2)	0.43 ± 0.26	10 (31.2)	0.45 ± 0.25
> 0 to < 40	37 (39.8)	0.36 ± 0.19	16 (50.0)	0.55 ± 0.63
≥ 40	14 (15.0)	0.37 ± 0.16	6 (18.8)	0.45 ± 0.21
Caffeine (mg/day)*				
0	35 (37.6)	0.35 ± 0.23	3 (9.4)	0.39 ± 0.19
< 300	48 (51.6)	0.41 ± 0.21	19 (59.4)	0.44 ± 0.22
≥ 300	10 (10.8)	0.48 ± 0.21	10 (31.2)	0.63 ± 0.76
Alcohol* (drinks/week)				
0	26 (28.0)	0.45 ± 0.29	5 (15.6)	0.64 ± 0.91
1–3	64 (68.8)	0.38 ± 0.19	17 (53.1)	0.41 ± 0.18
≥ 4	3 (3.2)	0.29 ± 0.09	10 (31.3)	0.55 ± 0.37

MET, metabolic equivalence.

a Mean FSH for days −5 to 1 calculated for cycles (vs. woman basis). Data from Women’s Reproductive Health Study, California Department of Health Services.

*p < 0.05 for test of independence between smoking status and covariate.

**Table 2:** Adjusted^a^ difference in FSH metabolic parameters by smoking level and 95% CIs.

	Cigarettes/day^b^		
Parameter and cycle days^c^	None (n = 186) Intercept	≤ 9 (n = 49) β (95% CI)	≥ 10 (n = 48) β (95% CI)
FSH slope			
−7 to −1	0.02	−0.02 (−0.04 to 0.00)	0.02 (−0.003 to 0.04)
−5 to 1	0.04	−0.02 (−0.05 to 0.01)	0.02 (−0.015 to 0.06)
−3 to 1	0.09	−0.04 (−0.10 to 0.01)	0.02 (−0.04 to 0.07)
Mean daily FSH			
−7 to −1	0.31	−0.03 (−0.12 to 0.06)	0.09 (−0.01 to 0.20)
−5 to 1	0.37	−0.06 (−0.17 to 0.05)	0.12 (0.00 to 0.24)
−3 to 1	0.40	−0.08 (−0.21 to 0.04)	0.14 (0.003 to 0.28)
−3 to 3	0.50	−0.08 (−0.22 to 0.06)	0.10 (−0.06 to 0.26)
1 to 5	0.68	−0.09 (−0.29 to 0.10)	0.006 (−0.22 to 0.23)

a Adjusted for age, race, pregnancy history, BMI, and alcohol and caffeine consumption in mixed models for repeated measures, with weighting of FSH parameter by proportion of nonmissing within the window.

b Smoking as reported during cycle starting with day 1; n is for the parameter with the largest numbers. These vary by a few cycles because of missing data; one outlier was excluded.

c Days are counted with first bleed day of a cycle numbered as day 1. Data from Women’s Reproductive Health Study, California Department of Health Services.

**Table 3:** Adjusted^a^ difference and 95% CIs in steroid hormone metabolite parameters by smoking level.

	Cigarettes/day		
Hormone parameter	None (n^b^ = 1,313) Intercept	≤ 19 (n = 117) β (95% CI)	≥ 20 (n = 25) β (95% CI)
Estrogen (ng/mg creatinine)			
Baseline	24.9	3.9 (0.06 to 7.6)	5.7 (−3.5 to 14.8)
Total FP	584.6	6.8 (−66.8 to 80.3)	29.1 (−159.6 to 217.8)
Daily average FP	39.0	1.2 (−3.1 to 5.5)	4.3 (−6.3 to 14.9)
Peak to periovulatory	70.4	−0.3 (−10.0 to 9.4)	−11.5 (−32.7 to 9.7)
Progesterone (μg/mg creatinine)			
Baseline	0.54	−0.03 (−0.14 to 0.08)	0.21 (−0.04 to 0.47)
Total LP	56.7	−1.7 (−9.4 to 5.9)	−15.5 (−32.2 to 1.1)
Daily average LP	4.66	−0.17 (−0.80 to 0.47)	−1.34 (−2.72 to 0.04)
Peak LP	6.64	−0.36 (−1.35 to 0.63)	−1.63 (−3.65 to 0.40)

Abbreviations: FP, follicular phase; LP, luteal phase.

a Adjusted for age, race, education, prior pregnancies, caffeine, and MET score (exercise level).

b n indicates the parameter with the largest numbers (e.g., follicular-phase estrogen and luteal-phase progesterone); others vary because of missing data, with peaks having smaller n values by 260–300 cycles overall. Data from Women’s Reproductive Health Study, California Department of Health Services.
